# Development and Pilot Testing of a Dispensing Protocol on Emergency Contraceptive Pills for Community Pharmacists in Belgium

**DOI:** 10.3390/pharmacy10030058

**Published:** 2022-06-01

**Authors:** Michael Ceulemans, Marieke Brughmans, Laura-Lien Poortmans, Ellen Spreuwers, Julie Willekens, Nele Roose, Isabelle De Wulf, Veerle Foulon

**Affiliations:** 1 Clinical Pharmacology and Pharmacotherapy, Department of Pharmaceutical and Pharmacological Sciences, KU Leuven, 3000 Leuven, Belgium; nele.roose@kuleuven.be (N.R.); veerle.foulon@kuleuven.be (V.F.); 2 Teratology Information Service, Netherlands Pharmacovigilance Centre Lareb, 5237 MH Hertogenbosch, The Netherlands; 3 L-C&Y, Child and Youth Institute, KU Leuven, 3000 Leuven, Belgium; 4 Faculty of Pharmaceutical Sciences, KU Leuven, 3000 Leuven, Belgium; mariekebrughmans@hotmail.com (M.B.); lauralien.poortmans@gmail.com (L.-L.P.); ellen.spreuwers@outlook.be (E.S.); willekens98@gmail.com (J.W.); 5 National Pharmacists Association, 1000 Brussel, Belgium; isabelle.dewulf@apb.be

**Keywords:** emergency contraception, morning-after pill, postcoital contraceptives, levonorgestrel, ulipristal, community pharmacy services, pharmacists, women’s health, women’s health services, counseling

## Abstract

Community pharmacists in Belgium frequently dispense emergency contraceptive pills (ECPs). However, variable and insufficient counseling practices exist across pharmacies, highlighting the need for standardization and quality improvement strategies. The aim of this project was to develop and test an ECP dispensing protocol for pharmacists. An ‘experience-based’ co-design approach involving academic and practicing pharmacists was applied, followed by a 4-month test period and interviews to assess users’ experiences. In total, eight geographically dispersed pharmacies participated. Pharmacists (*n* = 15) reached a consensus on most items to be included in the protocol, which was subsequently tested in seven pharmacies, with overall 97 registered ECP conversations. Pharmacists considered the protocol complete but felt that not all items should be mentioned/questioned during all conversations. They suggested only subtle modifications to be made prior to delivering a final protocol ready for nationwide distribution. Despite attributing positive effects to having a protocol, no single pharmacist ‘actively’ used it at-the-counter but used it instead as a ‘checklist’ after the encounter. Pharmacists found that the paper-based format of the protocol hindered protocol-based dispensing. Future research is needed to provide evidence on the actual benefits of protocol application, as well as to identify factors influencing the implementation of ECP dispensing using a software-integrated protocol.

## 1. Introduction

According to the World Health Organization, emergency contraception (EC) refers to “methods of contraception that can be used to prevent pregnancy after sexual intercourse” [1]. The use of EC can be needed after unprotected intercourse, contraceptive failure, incorrect use of contraceptives, and sexual assault in the absence of contraception use [1]. The currently available, licensed EC methods in Belgium are the copper intrauterine device (IUD) and the emergency contraceptive pills (ECPs), containing levonorgestrel (LNG) or ulipristal (UPA). Recently, the LNG-containing IUD has shown to be non-inferior to the copper IUD as EC but can only be used ‘off-label’ for this indication [2]. ECPs should be used within 3 (LNG) or 5 days (UPA) after unprotected sexual intercourse; however, the sooner they are used after intercourse, the more effective they are [3,4].

Despite being less effective as EC [5], ECPs are more frequently dispensed in community pharmacies in Belgium than IUDs, probably because of their high ease of use [6]. It is estimated that each day, ECPs are dispensed in approximately 1/10th of all pharmacies in Belgium. From an individual and public health perspective, it is vital that ECPs are used correctly to prevent unintended pregnancies. In Belgium, ECPs can only be purchased (over-the-counter (OTC)) in community pharmacies. The fact that ECPs cannot be bought in supermarkets or grocery shops emphasizes the important front-line role of pharmacists. In September 2020, the counseling role of Belgian pharmacists on this topic further increased when a new law came into effect, allowing pharmacists to dispense ECPs with reimbursement, without the need for a doctor’s prescription. In fact, ECPs were the first medicines for which Belgian pharmacists were given this responsibility, marking a huge milestone for the profession. The new legislation made a doctor’s visit meaningless, at least from a financial point of view, for patients requiring an ECP and willing to benefit from direct reimbursement in the pharmacy. This further increased the likelihood of pharmacists being the only healthcare professional (HCP) patients see when seeking EC.

Unfortunately, anecdotical reports and preliminary findings have shown that ECP counseling in the Belgian pharmacy setting does not always meet the required quality. In addition, counseling differences across pharmacies exist, underscoring the need for standardization and quality improvement strategies [7,8]. Varying practices and gaps in information gathering and provision of advice on ECPs have also been observed in other developed countries such as Australia, United Kingdom, Germany, and Canada [9,10,11,12,13,14,15].

To standardize ECP dispensing and facilitate consistency in the provision of pharmacy-based ECP services, protocols/checklists have been developed in some countries (e.g., Australia, Switzerland, Germany, and the United Kingdom) [16,17,18,19]. According to a recent publication from Germany [13], the application of an ECP ‘checklist’ by pharmacists led to a “higher questioning score” (i.e., higher number of questions addressed by pharmacists), which was associated with achieving the appropriate outcome (i.e., dispensing the correct product in that specific scenario). High adherence to the ECP protocol was also observed among Swiss pharmacists who adequately counseled simulated patients requesting an ECP [20]. This is in contrast to another study in Belgium showing that pharmacists’ questioning score and information gathering during reproductive health services did not differ after having followed a blended training program [21]. These observations provide evidence on potential benefits and opportunities of protocol-based counseling on ECPs. Unfortunately, an ECP dispensing protocol did not yet exist for community pharmacists in Belgium at the start of this project.

Given the increasingly important role of community pharmacists in EC counseling, the potential benefits but current lack of an ECP dispensing protocol, and the suboptimal quality of ECP counseling in Belgium, efforts were needed to pursue and contribute to the standardization, optimization, and quality assurance of EC(P) counseling. Therefore, a project was set-up, in collaboration with the National Pharmacists Association (APB), to develop and pilot test an ECP dispensing protocol for community pharmacists in Belgium.

## 2. Materials and Methods

### 2.1. Study Design and Sample

The project consisted of three consecutive parts, i.e., (1) development of an ECP dispensing protocol; (2) pilot testing of the ECP dispensing protocol; and (3) protocol optimization based on users’ experiences. First, an ‘experience-based’ co-design approach involving pharmacy practice researchers and practicing pharmacists employed in community pharmacies in Flanders, Belgium (i.e., pilot pharmacies), was used to develop a ‘test’ version of the ECP protocol. Second, this ‘test’ protocol was tested in pharmacy practice by the same set of pilot pharmacies. Third, users’ experiences were collected via semi-structured interviews with pharmacists employed in the pilot pharmacies and subsequently used for optimization purposes to result in a final protocol, ready for nationwide implementation. By adopting this ‘multifaceted’ approach, we aimed to consider the preferences and experiences of practicing pharmacists and to maximize the protocol’s utility and application potential upon implementation.

The project took place between October 2020 and March 2021, i.e., during the second wave of the COVID-19 pandemic. The selection of pilot pharmacies was performed via convenience sampling, hence recruiting pharmacists with a large interest to participate. As an inclusion criterion, pilot pharmacies needed to dispense at least 40 boxes of any type or brand of ECP each year, ensuring sufficient experience with the topic of interest and ample testing opportunities during the pilot period. Information on the project was distributed by the national and local professional pharmacy organizations through digital newsletters.

All participating pharmacists provided written informed consent prior to study initiation. Ethical approval was obtained from the Ethics Committee Research UZ/KU Leuven (MP016086; 22 September 2020).

### 2.2. Protocol Development through Co-Design

To develop the ‘test’ protocol, an online focus group discussion with pilot pharmacists was organized as a ‘co-design’ event in October 2020. Due to the COVID-19 pandemic, we were obliged to use an ‘online’ format. The group discussion lasted two hours and was moderated by researchers M.C. and V.F. Four students of the Master in Pharmaceutical Care also attended the focus group (M.B., LL.P., E.S., and J.W.), as well as representatives of the National Pharmacists Organization (I.D.W.) and Sensoa, the regional expertise center on sexual and reproductive health.

The ‘co-design’ event started with a brief presentation by the moderators of some project information, followed by an open-minded discussion with all attendees focusing on two aspects: (1) which questions pharmacists would ask when someone requests EC and (2) which information pharmacists would provide when dispensing ECPs. The moderators invited all attendees to provide input and suggestions on both questions. In the second part of the meeting, a draft version of an ECP protocol, initially created by the researchers based on personal insights, pharmacy experiences, and literature [16,17,18], was shared with the attendees to further provoke the discussion in relation to the suggestions collected earlier-on in the meeting. The ‘co-design’ event eventually resulted in the development of the ‘test’ protocol. The meeting was recorded and transcribed verbatim.

### 2.3. Pilot Testing

As soon as the ‘test’ protocol was available, a four-month test period in the pilot pharmacies commenced (i.e., November 2020–February 2021). Pilot pharmacists were asked to motivate their team, including pharmacy-technicians (PT), to use the protocol during each conversation on ECP. Pharmacists were also invited to register baseline data after each ECP conversation using a predefined, paper-based registration document. While pilot pharmacists were neither trained by the researchers on the components nor on the actual application of the protocol in practice, some documentation with theoretical concepts on ECPs was available to all pharmacists.

### 2.4. Protocol Optimization Based on Users’ Experiences

At the end of the pilot period, pharmacists were invited to participate in an online, semi-structured interview to gain insight into pharmacy staff’s experiences with using the ‘test’ protocol in practice. It was our goal to interview at least one pharmacist from each pilot pharmacy. Interviews were carried out in March 2021 by a female Master student, E.S., using a topic guide consisting of questions exploring pharmacy staff’s experiences with the content of the protocol (i.e., feasibility of protocol items and occurrence of any missing items) and applying the protocol in daily practice. The obtained insights were used to optimize the ‘test’ protocol and to deliver a final version. The interviews were audio-recorded and transcribed verbatim.

### 2.5. Data Analysis

The qualitative data obtained during the ‘co-design’ event were descriptively summarized and presented as a narrative text, including representative statements. The interview data were analyzed by two researchers using an inductive, thematic approach in NVivo and Excel and according to the Qualitative Analysis Guide of Leuven (QUAGOL) [22]. The registration documents were quantitatively analyzed using absolute counts (n) and percentages.

## 3. Results

### 3.1. Description of the Participants

In total, eight community pharmacies were included as the pilot pharmacies. These pharmacies were geographically distributed across Flanders, Belgium, with four of the five Dutch speaking regions in Belgium being represented. During the ‘co-design’ event, 15 community pharmacists of different age groups attended (12 women and 3 men).

### 3.2. Protocol Development through Co-Design

During the ‘co-design’ event, pharmacists agreed that the protocol ideally consisted of four parts and supported pharmacists in four actions, i.e., (1) the ‘intake’ with a list of questions exploring the actual situation; (2) the ‘decision making’ based on the collected/summarized information and explaining this to the patient; (3) the ‘informative’ part, providing patient-specific advice; and (4) asking for feedback (i.e., checking if the patient understood all information correctly) and for questions or concerns.

With regard to the ‘intake’, pharmacists agreed that the first/important questions were (1) for whom the ECP was requested and (2) when the (unprotected) sexual intercourse took place. Pharmacists further agreed that upon identification, a clear distinction should be made between women using or not using (hormonal) contraception. In case of contraception use, pharmacists confirmed it should be questioned which contraceptive the woman is using, what happened with her contraceptive and when she had her last hormone-free interval. If no (hormonal) contraceptive was used, they found it vital to ask the woman about the timing of her last menstrual period and the average duration of her cycle. Other questions that were considered important by pharmacists related to medication use in the last 4 weeks and previous ECP intake. Depending on the patient’s situation, questions related to breastfeeding and sexually transmitted diseases (STDs) could be used, according to some pharmacists, but were generally not considered pivotal. The ‘open’ question “Why do you think you need EC?” was suggested and supported by some pharmacists, although opinions on appropriateness and preferences to use this question widely varied.

Further, pharmacists agreed that after the intake and acquiring essential information on the specific situation/context, the protocol should support pharmacists in outweighing possible choices and discussing these with the patient. For example, the use of EC(P) may or may not be needed, and/or a referral to a medical doctor might be (more) appropriate. Pharmacists felt that this second part of the protocol was ideal for this purpose, including aspects related to the price/reimbursement of ECP and the registration of ECP dispensing in pharmacy software (if possible, on the name of the ECP user).

With regard to the ‘informative’ part, pharmacists totally agreed on the inclusion of the following items in the protocol to support patient counseling: (1) ‘mechanism of action’, i.e., the fact that ECPs postpone ovulation; (2) the ‘advice’ to take the single pill as quickly as possible and to take a new pill in case of vomiting within 3 h after the intake; (3) information on potential side effects, such as earlier or later occurrence of menstruation; and (4) ‘specific information’, including (a) an ECP does not provide protection against upcoming sexual intercourse, (b) how to continue the use of contraception, and (c) the required time to use condoms until hormonal contraception is effective (again). Most pharmacists also agreed to tell patients that ECPs are not 100% effective. In contrast, two items were identified on which pharmacists disagreed about including in the protocol, i.e., (1) to tell patients that an ECP is not an ‘abortion’ pill and (2) that ECPs have no proven effect on women’s future fertility. Pharmacists were hesitant about the necessity to (always) mention these items when dispensing an ECP. Both items were therefore included in italics in the ‘test’ protocol. Overall, pharmacists stressed the importance to find the right balance between providing sufficient and too much information to make sure that patients remember all relevant details. Pharmacists agreed that written information may be beneficial. They were convinced that providing a patient leaflet at the time of delivery, or referring patients to reliable online sources, would be an added value.

### 3.3. Pilot Testing

During the pilot period, the ‘test’ protocol was used in seven of the eight pilot pharmacies (one pharmacy did not use the protocol, as in the multitude of documents that were sent to the pharmacies as part of this project (i.e., EC(P) guideline with corresponding flow chart, patient leaflets, and dispensing protocol), their attention for the protocol was lost).

In total, 97 ECP conversations (median per pharmacy: 8, range: 0–46) were registered, with 91 ECPs dispensed. Pharmacists did not always register information for all variables during the ECP conversations (see Supplementary Table S1 for more details). Depending on the specific variable, missing data were observed for 1 (1%) to 41 (42%) of the conversations, including the timing of sexual intercourse (10%), indication/reason for requesting ECP (9%), and concomitant use of contraception (6%).

### 3.4. Pharmacy Staff’s Experiences with the Content of the Protocol

In total, eight pharmacists participated in the interviews, of which six women and two men were all employed across the different participating pilot pharmacies.

In general, pilot pharmacists did not find any ‘intake’ question in the ‘test’ protocol (totally) redundant. However, pharmacists confirmed that not all questions should always be asked during ECP conversations. Pharmacists indicated that they prefer to use the protocol and items dynamically and depending on the specific situation. Moreover, opinions on the open question “Why do you think you need EC?” still varied; as it was not clear whether this should be included in the protocol or asked ‘as such’ in practice, this question was finally removed.

*“The question ‘why do you think you need EC’? Well, not every patient is ‘ready’ to immediately elaborate on the actual situation.”* (Male pharmacist, Pharmacy 7)

*“I prefer the question ‘why do you think you need EC’? instead of asking all items myself, as you may learn a lot about the patient herself. In response to this question, patients often spontaneously tell quite a lot, which makes it easier to respond to their story and ask additional questions if needed.”* (Female pharmacist, Pharmacy 1)

With regard to the ‘decision’ part, not all pharmacists reported having discussed the potential EC(P) options with patients or having explained their decision(s) about whether or not to deliver EC and, if so, what type of EC(P) was preferred. Nevertheless, pharmacists felt it relevant and justifiable to keep this second part in the final protocol. Moreover, opinions/practices also differed regarding the spontaneous provision of information on the price of ECP prior to payment. Given that such information is indispensable according to some pharmacists, certainly in case of dispensing UPA when LNG is also an option, the item referring to price/reimbursement was kept in the final protocol.

*“The question from patients about the price of an ECP is a question which actually pops up almost always. Hence, and especially if you want to dispense ulipristal, you should almost always talk about the price of the ECP.”* (Male pharmacist, Pharmacy 3).

With regard to the ‘information’ part, pharmacists agreed on the inclusion of protocol items related to the general topics of ‘mechanism of action’, ‘use’, ‘potential side effects’, and ‘information on the use of contraception/condoms’. However, pharmacists did not agree on the necessity to spontaneously mention some specific items during all conversations. This was the case for (no) effect of ECPs on future fertility, an ECP not being an ‘abortion’ pill nor providing protection against STDs, and the advice for STD screening. Since these items could be part of some conversations on EC(P), for example, upon identification of a patient’s distress regarding one of these topics, these items were kept in italics in the final protocol, except for the word ‘abortion’, which was considered inappropriate to use and was changed into ‘not interrupting an ongoing pregnancy’.

*“I don’t use the word ‘abortion’. Instead, I say that the ECP prevents fertilization but that it does not interrupt an ongoing pregnancy.”* (Female pharmacist, Pharmacy 1).

Pharmacists cited that mentioning all items or not may actually depend on the specific patient asking for an ECP. For example, it may be difficult during a conversation with a rushed patient or male customer who is ignorant about the woman’s menstrual cycle or the type of contraception used. Finally, some pharmacists confirmed that patients should not be overwhelmed with too much information, as they probably will not remember everything. Providing a leaflet may be a better alternative for conveying all relevant information.

### 3.5. Pharmacy Staff’s Experiences towards Using the Protocol in Practice

#### 3.5.1. Actual Protocol Utilization in Practice

Overall, all pharmacists reported having used the protocol solely after ECP dispensing as a ‘checklist’ to ensure that all items were questioned/discussed. In other words, the ‘test’ protocol was not used at-the-counter during conversations with patients. Pharmacists considered the use of a paper-based protocol during counseling a real barrier. Several factors contributing to this experience were identified, such as (1) hindering the communication/dialogue with patients; (2) making ECP dispensing less spontaneous/more artificial (i.e., using it as a rigid checklist, thereby only focusing on the ‘technical’ aspects of the request); (3) appearing unprofessional or ignorant to patients; and (4) giving patients the impression that seeking ECP is a ‘big deal’, which may potentially scare them off. Pharmacists felt that communication with patients in a human way is critical to create trust, which is especially important in case of a ‘sensitive’ topic, such as ECP counseling.

*“Grabbing a paper at the counter is a bit weird for patients, because then they realize that we are ‘cheating’, but when you look at the protocol on your computer, the patient does not realize it.”* (Female pharmacist, Pharmacy 1).

*“I find it a real threshold to use the paper-based protocol during the conversation with a patient. By using the paper, it seems like you don’t know the content very well, and it can look like a big deal for a patient, which will scare them off.”* (Female pharmacist, Pharmacy 5).

Most pharmacists acknowledged that they prefer a digital protocol rather than a paper-based one. However, if too many pop-ups appear, this would stop pharmacists from using a digital version as well. Pharmacists noted that a balance should be found during counseling between looking at the computer and the interaction (having eye contact) with patients.

#### 3.5.2. Pharmacy Staff’s Self-Reported Advantages of Having a Protocol

Several self-reported advantages of having an ECP protocol were identified. Overall, pharmacists felt that ECP conversations/dispensing were more comprehensive, complete, and structured. The observation that conversations with patients lasted longer was not considered negative. Some pharmacists also mentioned that thanks to the availability of the protocol, they more often referred patients to doctors, for example, to discuss the use of contraception. Finally, it was noted that having a protocol created more awareness about the theoretical and practical aspects of EC(P) dispensing in practice. According to pharmacists, using the protocol may be beneficial for patient care and counseling quality and enhance patient involvement and support.

*“Thanks to the protocol, the conversation with the patient lasted longer. People were longer inside the pharmacy, but it is not a negative aspect. On the contrary, it felt that more questions came from patients themselves. I found this a very positive experience and so did my colleague. She said to me ‘I really think this is an added value’.”* (Female pharmacist, Pharmacy 4).

*“The advantage is that you certainly question all aspects that you need to know and thus have complete information. You can then make a better decision.”* (Female pharmacist, Pharmacy 6).

*“We now work in a much more structured way. People get a lot more explanation. In the past, we used to ask ‘the emergency contraceptive pill? Are you taking something of protection? Or what happened?’, but we did not elaborate on it then. Now, with the protocol, we have a guideline for going more into detail.”* (Female pharmacist, Pharmacy 4).

## 4. Discussion

### 4.1. Main Findings

This project aimed to develop and test an ECP dispensing protocol for community pharmacists in Belgium. As part of the development phase, an ‘experience-based’ co-design approach involving academic and practicing pharmacists was applied. The protocol was subsequently tested in seven pilot pharmacies for 4 months (with ±100 ECP ‘test’ encounters) and eventually optimized based on users’ experiences collected during interviews.

During the development part, pharmacists agreed on the four parts of the ECP protocol (i.e., ‘intake’, ‘decision’, ‘advice’, and ‘feedback’), as well as on most individual items to be included in the ‘test’ protocol. After having used this protocol in practice, pharmacists reported that the protocol was complete and that no relevant items were actually missing. Although the items are generally quite similar to the ones included in the ECP protocols abroad [16,17,18], the structure of the Belgian protocol (i.e., four parts) is somewhat different.

Some pharmacists felt that some specific items should not (always) be spontaneously questioned or mentioned during ECP counseling. Therefore, in the final protocol, these ‘debatable’ items were mentioned in italics (see Supplementary Figure S1). This observation shows that pharmacists do not like to stick rigorously to a pre-defined list (‘checklist’), but they rather want to use a protocol or checklist with some ‘freedom’, depending on the actual situation or patient. Of course, we understand and even acknowledge that pharmacy conversations may differ, and that practitioners should be given the opportunity to deviate from a protocol in some cases. However, we believe that vital information required to make a sound decision (i.e., to dispense EC or not, and if so, which type of ECP) and/or items to adequately instruct patients on using ECP and follow-up contraception should be part of *all* ECP conversations. Nonetheless, the data collected during the pilot period show that information on some of the critical items was missing in quite a high number of conversations, underlining the need for further quality improvement in counseling. This finding is in line with an Australian study showing poor application of the ECP protocol (items) in practice [12]. In contrast, according to a recent study on ECP counseling, Swiss pharmacists asked 10.9 of the 11 EC assessment questions listed in the official protocol [20], marking a very high adherence to and successful implementation of the protocol/checklist in pharmacy practice.

Our ‘final’ protocol became available to all pharmacists in Belgium in Autumn 2021. Since its nationwide distribution, two important considerations with regard to the content of the protocol have been identified and deserve further attention. First, it may be appropriate to modify the order of questions in the ‘intake’. It would make more sense if pharmacists first ‘validate’ the ECP request (i.e., exploring whether EC is truly required or not), before asking about the actual timing of sexual intercourse (i.e., this question is irrelevant in the absence of an indication for EC). Second, a question to find out the patient’s weight/body-mass index (BMI) is missing in the protocol, potentially due to the uncertainty/inconsistency in the available ECP guidelines at the time of development of the dispensing protocol. However, a question on weight/BMI is highly relevant, as weight can affect the recommended dose of LNG (=3 mg) [23] and should therefore be added to the protocol. The adaptation of the dose in a function of weight has recently also been included in the revision of the ECP guideline in Belgium. Nonetheless, according to previous research, pharmacists may feel uncomfortable discussing body weight with patients [6].

With regard to the actual utilization of the protocol in practice, pharmacists indicated having used it solely *after* the delivery as a ‘checklist’ to ‘check’ if all items had been discussed, and thus, they did not actively use the protocol at-the-counter to support ECP deliveries. Thus, pharmacists had to rely on their memory during counseling to recall all protocol items, potentially explaining, at least to some extent, the degree of missing data in the registrations obtained during the pilot period. This was confirmed by previous studies on ECP counseling showing that if pharmacists (visibly) used a checklist, high(er) questioning scores were obtained and, eventually, a high(er) likelihood of achieving the appropriate outcome [13,20].

The interviews provided preliminary insights into potential reasons explaining why pharmacists did not ‘actively’ use the ECP protocol at-the-counter, impeding its successful implementation in practice. As the most important barrier, it was found that the paper-based format hindered pharmacists. The ‘final’ protocol has meanwhile become available as a pdf version, providing pharmacists the opportunity to save the protocol on their computer (desktop) and use it as such during counseling. Our findings show that protocol-based dispensing (of ECPs) is not yet common practice in the Belgian pharmacy setting. Still, pharmacists attributed several benefits to having an ECP protocol, both in terms of the quality of ECP conversations and patient care. This would be appropriate given the variable practices in ECP counseling in Belgium [7]. As soon as the ECP protocol is integrated in pharmacy software, studies are needed to identify barriers and facilitators influencing the implementation of protocol-based dispensing of ECPs in that way [24].

On top of that, it still remains unknown to what extent the application of the protocol will succeed in the standardization of ECP conversations and lead to better ‘outcomes’ in terms of counseling quality (e.g., appropriate decisions and correct and complete advice) and patient satisfaction with reproductive health pharmacy services. Such assessments are needed in the future [25,26], especially as previous research in Australia has shown that although the use of a written ECP checklist improved the quantity and consistency of patient assessment, it did not result in a higher frequency of appropriate decisions [27]. Ultimately, there is no evidence on the knowledge of Belgian pharmacists on EC nor on the knowledge and counseling preferences of the Belgian public regarding this topic. Such evidence is needed to identify any opportunities to optimize EC(P) counseling, and it is actually warranted given the recent observation of a lack of knowledge on reproductive and obstetric medication use among Belgian pharmacists [28].

### 4.2. Strengths and Limitations

The project has several strengths. First, the co-design approach involving practicing pharmacists in protocol development and optimization may have resulted in the release of a protocol, the content of which is in line with the preferences and views of end users. This co-design approach may also have increased the feasibility, utility, and acceptance of the ECP protocol. Second, the inclusion criterion of dispensing at least 40 boxes of ECPs annually ensured that only pharmacists with experience on the subject could participate. Third, the online focus group facilitated the inclusion of pharmacists employed in geographically diverse regions and (technically) ran very smoothly. This is in line with the findings of recent studies showing the feasibility of online approaches for focus group or consensus methodology [29,30,31]. Finally, this study provided the first insights into the experiences of pharmacists in Belgium with protocol-based dispensing of ECPs and identified barriers impeding its implementation in practice, upon which future studies can be further built.

Some limitations can also be addressed. First, only Dutch-speaking pharmacists were enrolled via convenience sampling, recruiting mainly highly motivated colleagues, which may have resulted in selection bias. It remains unknown to what extent the ECP protocol will be accepted by French-speaking pharmacists in Belgium and Dutch-speaking colleagues with less experience with and/or interest in this topic. Second, due to the COVID-19 pandemic and social restriction measures, fewer ECPs were requested/dispensed in the pilot pharmacies during the test period, limiting the possibilities to ‘test’ the protocol. To solve this limitation, the duration of the test period was extended (i.e., from 2 to 4 months). However, pharmacists acknowledged that not all ECP conversations that happened during the pilot period had been registered. Thus, the 97 registered conversations were without any doubt an underestimation of the actual number of encounters during the pilot period. For some variables, a high number of missing data was observed. It is unknown whether pharmacists did not register this information because of not having collected it during the encounters (due to the lack of protocol application at-the-counter) or whether the information had already been forgotten at the time of registration (which may have occurred after some other patient conversations). The uncertainty concerning the accuracy of recall when completing the registration document should also be considered. Finally, the ‘positive effects’ of using the ‘test’ protocol were collected during interviews and were not objectively observed in real-life, limiting the validity of these findings.

## 5. Conclusions

An ‘experience-based’ co-design approach involving academic and practicing pharmacists and followed by a four-month test period resulted in a ECP dispensing protocol for community pharmacists in Belgium. Pharmacists considered the protocol complete but felt that not all protocol items should be mentioned/questioned during all ECP conversations. They suggested only subtle modifications to be made after the test period and prior to delivering a final protocol ready for nationwide distribution. Despite attributing positive effects to having a protocol, no single pharmacist ‘actively’ used it at-the-counter but used it instead as a checklist after the encounter. Pharmacists found that the paper-based format of the protocol hindered protocol-based dispensing of ECPs by pharmacy staff. Future research is needed to provide evidence on the actual benefits of ECP protocol application in terms of counseling quality and patient satisfaction, as well as to identify factors influencing the implementation of ECP dispensing using a software-integrated protocol.

## Data Availability

Not applicable.

## Supplementary Materials

The following are available online at https://www.mdpi.com/article/10.3390/pharmacy10030058/s1. Table S1: Overview of the registered variables of the ‘emergency contraception’ conversations during the pilot period; Figure S1: English version of the emergency contraception protocol (Autumn 2021).
