# Supplementary material for: Development and Pilot Testing of a Dispensing Protocol on Emergency Contraceptive Pills for Community Pharmacists in Belgium
Source: Pharmacy (Basel). 2022 Jun 1;10(3):58. doi: 10.3390/pharmacy10030058 (PMC9228420; doi:10.3390/pharmacy10030058)
Supplement: Supplementary file 1 [file pharmacy-10-00058-s001.zip › pharmacy-1720824-supplementary for xml.pdf]

Supplementary Table S1: Overview of the registered variables of the 'emergency contraception' conversations during the pilot period (N=97).

Variable	N (%)
Person who requested the ECP*	
Patient herself	70 (72.2)
Partner	15 (15.5)
Other	6 (6.2)
Missing	6 (6.2)
Age of the ECP* user	
18-25 years	28 (28.9)
26-35 years	22 (22.7)
>35 years	5 (5.2)
<18 years	1 (1.0)
Missing	41 (42.3)
Concomitant use of contraception	
None	71 (73.2)
Combined contraceptive pill	9 (9.3)
Condoms	6 (6.2)
Progestogen-only pill (POP)	2 (2.1)
Other	2 (2.1)
Contraceptive injection	1 (1.0)
Missing	6 (6.2)
Timing sexual intercourse	
< 24 hours	47 (48.5)
24-72 hours	36 (37.1)
72-120 hours	4 (4.1)
Missing	10 (10.3)
Type of ECP* dispensed	
Ulipristal acetate	43 (44.3)
Levonorgestrel	47 (48.5)
None	6 (6.2)
Missing	1 (1.0)
Indication for requesting ECP*	68 (70.1)
No use of contraception	9 (9.3)
Two or more forgotten pills	4 (4.1)
Other	3 (3.1)
Wrong use of contraception	2 (2.1)
One forgotten pill	1 (1.0)
Hormone-free interval too long	1 (1.0)
Intake POP too late	9 (9.3)
Missing	
Referral to / reason of referral to a doctor	60 (61.9)
No	25 (25.8)
Initiation / adjustment of contraception	6 (6.2)
Potential risk of sexual transmitted disease	4 (4.1)

Other	2 (2.1)
Suspicion of pregnancy	1 (1.0)
Consideration of an IUD** as emergency contraception	4 (4.1)
Missing	
Registration of the dispensed ECP* on the patient's name	47 (48.5)
Yes	33 (34.0)
No	11 (11.3)
Missing	
Provided a patient leaflet	50 (51.5)
Yes	45 (46.4)
No	2 (2.1)
Missing	

Results are expressed as absolute numbers (%). 'Missing' refers to the missing values for the corresponding variable. Numbers may exceed 100% as several response options were possible for a specific variable. *ECP = emergency contraceptive pill; **IUD = intrauterine device

Supplementary Figure S1: English version of the emergency contraception protocol (Autumn 2021). Part 1: exploring the question for emergency contraception; Part 2: summary and decision; Part 3: counseling on the use of an emergency contraceptive pill; Part 4: closing the conversation.

1

Part 1: Exploring the question for emergency contraception

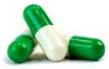

Investigate the reason for requesting emergency contraception.

Gather contextual information and explore the person's knowledge with regard to the use of emergency contraception – ask OPEN QUESTIONS!

- ✓ **For whom** the emergency contraceptive pill has been requested?
- ✓ **When** did the sexual intercourse take place (you are worried about)?
- ✓ Does the woman use (hormonal) **contraception**?

  YES: Which method / type of pill (e.g., 21+7, …)? What's the brand name?
  YES: What went wrong with the use of contraception? When?
  How many pills were / have been forgotten?
  YES: When was the last pill-free period / stop week?

  NO: When was the last menstrual period?
  What is the average duration of the menstrual cycle?

- ✓ Which **other medicines** does the woman use?
- ✓ What is her **previous experience** with the use of the emergency contraceptive pill?
- ✓ *When breastfeeding: when did the woman give birth? How many times a day she is breastfeeding? Does the woman express milk?*

Part 2: Summary and decision*

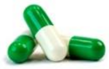

Summarize the gathered information.

Indicate the best option(s) in this situation:

- ✓ Emergency contraception is needed;
- ✓ Emergency contraception is not needed; give information/education;
- ✓ Referral to a doctor (e.g., for an emergency intrauterine device (IUD) and/or consultation regarding sexually transmitted diseases).

<u>When emergency contraception is needed:</u>

Choose between an IUD and an emergency contraception pill (levonorgestrel/ulipristal) in function of patient's characteristics / patient preferences / situation.

You could mention the price of both types of emergency contraceptive pill.

Inform the patient that she can benefit from reimbursement for an emergency contraceptive pill, and that you will therefore need to register the patient's ID (reimbursement can only be applied in case of delivery to a woman who is insured).

* More information about the actual choice of emergency contraception can be found in the accompanying document and flowchart. When making a choice, please pay attention to the concomitant use of CYP3A4 inducers (St. John's wort, carbamazepine,…), the body-mass index (BMI),…

3

Part 3: Counseling on emergency contraceptive pill use (ECP)

MECHANISM OF ACTION

- ✓ The ECP will **postpone the ovulation** and **prevents fertilization**.
- ✓ The ECP does not prevent implantation and does not interrupt a pregnancy.
- ✓ The ECP does only protect against previous sexual intercourse and thus, **does not provide protection until the following menstruation**.
- ✓ The ECP does not protect against sexually transmitted diseases (STDs).
- ✓ *The ECP has no influence on a woman's (future) fertility.*

ACTUAL USE

- ✓ Take **1 tablet AS SOON AS POSSIBLE**.
- ✓ **In case of vomiting within 3 hours** after the intake: **take a new tablet**.
- ✓ *When breastfeeding: discuss which measures need to be taken.*

SIDE EFFECTS

- ✓ **Possible**, for example nausea, vomiting, headache, menstrual complaints.
- ✓ Earlier (rather LNG) or later occurrence of menstruation (rather UPA).

FOLLOW-UP / COMPLIANCE

- ✓ Discuss **how to continue the use of contraception** + **duration condom**.
- ✓ For more information about contraception: refer to a doctor or the following web site (www.allesoverseks.be).
- ✓ The ECP is **not 100% effective** and less effective than regular contraception; the use of a pregnancy test is possible 14 days after unprotected sexual intercourse.
- ✓ *Advise the use of condoms for the prevention of STDs.*
- ✓ *Refer to a doctor for a STD consult after a risky contact.*

4

Part 4: Closing the conversation

Ask feedback to learn if the person has understood the information.

Check **which questions or concerns the person still has** (e.g., any influence of the ECP on future fertility, influence of the ECP on an fertilized egg, …).

Provide a **patient leaflet**.
